# First‐Line Treatment of Icaritin and Thalidomide in a Patient With Hepatocellular Carcinoma With PR: A Case Report

**DOI:** 10.1002/cnr2.70136

**Published:** 2025-03-04

**Authors:** Zaiyong Jin, Fei Zhou, Zhuo Wang, Hongji Li

**Affiliations:** ^1^ Department of Hepatological Surgery Jilin City Central Hospital Jilin China; ^2^ Medical Image Central, Jilin City Central Hospital Jilin China; ^3^ Department of Abdominal Imaging Diagnostic Jilin City Central Hospital Jilin China; ^4^ Department of Traditional and Western Oncology Jilin City Central Hospital Jilin China

**Keywords:** combination therapy, icaritin, late‐stage hepatocellular carcinoma, partial remission, thalidomide (TLD)

## Abstract

**Background:**

Hepatocellular carcinoma (HCC) remains a significant global health burden, with unmet clinical needs despite the availability of multiple therapeutic options.

**Case:**

We present the case of an 85‐year‐old male diagnosed with HCC and bilateral lung metastases following hepatectomy. The patient responded favorably to treatment with icaritin and thalidomide, which resulted in a reduction in alpha‐fetoprotein (AFP) levels and tumor size. This treatment achieved partial remission, with a progression‐free survival (PFS) of 24 months and an overall survival (OS) of 33 months. Unfortunately, the patient ultimately passed away due to a cerebral infarction unrelated to cancer progression.

**Conclusion:**

This case underscores the potential of icaritin as a therapeutic option for HCC patients with compromised health status. The combination of icaritin and thalidomide demonstrated promising efficacy in this real‐world scenario. Multidisciplinary combination treatment strategies incorporating icaritin merit further exploration, given its immunomodulatory effects and favorable safety profile.

## 1 Introduction

Hepatocellular carcinoma (HCC) is a prevalent malignant tumor and ranks as the second leading cause of cancer‐related mortality worldwide [1]. In China, it accounts for approximately 55% of new HCC cases annually [2]. Early detection is challenging, as most patients have underlying liver disease with subtle symptoms, often resulting in late‐stage diagnoses that preclude optimal surgical intervention. Metastatic progression is common following localized treatments, making systemic therapy critical for advanced HCC. Despite advances in innovative treatment protocols and systemic therapies, approximately 20%–30% of patients are still diagnosed at late stages, leading to deteriorating health and complications. Standard therapies, including molecularly targeted agents and immune‐based combinations, often produce suboptimal outcomes due to efficacy and safety concerns. Consequently, there is an urgent need for safer and more effective treatments to improve survival and quality of life for HCC patients.

Icaritin is a potent small‐molecule immunomodulator with distinct effects on the immune system. It primarily acts by suppressing pro‐inflammatory signaling pathways, activating both innate and adaptive immunity, and optimizing the tumor immune microenvironment for enhanced antitumor activity. Icaritin inhibits the IL‐6/JAK2/STAT3 pathway by blocking the phosphorylation of JAK2 and STAT3, which suppresses downstream gene expression, prevents STAT3 nuclear translocation, and reduces tumor cell proliferation. This inhibition also correlates with increased levels of IFN‐γ. Additionally, icaritin targets the NF‐κB pathway by binding to MyD88/IKKα, disrupting the TLR‐MyD88‐IKK‐NFκB signaling cascade, and lowering the production of TNF‐α and IL‐6. Furthermore, icaritin enhances the cytotoxic response of CD8+ T cells, restoring their ability to secrete IFN‐γ. Elevated IFN‐γ levels activate dendritic cells and macrophages, improving antigen presentation and strengthening innate immunity. Importantly, icaritin reduces the population of immunosuppressive cells, such as myeloid‐derived suppressor cells (MDSCs), and suppresses PD‐L1 expression, thereby revitalizing the tumor‐eradicating functions of T cells and promoting adaptive immunity [3, 4, 5, 6, 7, 8, 9, 10].

Phase III clinical trials of icaritin have demonstrated its definitive efficacy and significant survival benefits for advanced HCC patients, along with good safety and tolerability. Icaritin shows comparable efficacy and notable safety advantages over existing first‐line standard treatments [11]. Regarding combination therapy, icaritin has potential synergy with the immune system in the target area. The use of antiangiogenic agents can counteract the immunosuppressive effects of vascular endothelial growth factor (VEGF), regulate the tumor vascular system by activating effector T cells, and enhance immune cell infiltration, thereby achieving synergistic effects [12]. Additionally, icaritin may boost the cytotoxic effects of thalidomide on multiple myeloma cells, increasing cytotoxicity from 25% to 50% [13].

We present a patient with advanced postoperative HCC and pulmonary metastasis who received icaritin and thalidomide as first‐line treatments. The patient achieved a partial response (PR) and a complete response (CR) lasting over a year, demonstrating satisfactory efficacy and safety.

## 2 Case Presentation

An 83‐year‐old male, originally from China, had a liver lesion detected in August 2012. Ultrasound and CT scans revealed a mass in the right lobe of the liver, however, the patient exhibited no significant symptoms, such as abdominal pain or bloating. Due to his advanced age and associated risks, no treatment was initiated. During subsequent follow‐ups, the liver mass enlarged significantly, leading to liver cancer resection in June 2014. Postoperative pathological examination confirmed HCC (pTXN0M0).

On December 15, 2014, the patient initially presented to Jilin City Central Hospital. He had a 40‐year history of smoking and drinking and presented with cough, sputum production, fatigue, and abdominal distension. A CT scan revealed recurrent liver nodules and bilateral lung metastases. His medical history included: (1) myocardial infarction over 20 years ago, (2) lacunar cerebral infarction for 15 years, (3) radical surgery for colon cancer in 2002 with four cycles of adjuvant chemotherapy (regimen unknown), (4) chronic hepatitis C for 12 years, with an HCV RNA level of 17 400 copies/mL in August 2014, (5) an untreated liver lesion detected in August 2012 due to lack of symptoms and advanced age, and (6) radical hepatectomy in June 2014 for an enlarged liver mass, confirmed postoperatively as HCC. The patient's vital signs were relatively stable, with an Eastern Cooperative Oncology Group Performance Status (ECOG‐PS) of 3 and a Child‐Pugh liver function score of B, 8. Laboratory results indicated elevated tumor markers: AFP > 2000 ng/mL, CEA 2.19 ng/mL, cytokeratin 17.21 ng/mL, and CA19‐9 14.43 μ/mL. The patient was diagnosed with stage D according to the Barcelona Clinic Liver Cancer (BCLC) classification, corresponding to stage IV in the latest China Liver Cancer Staging (CNLC), with intrahepatic and bilateral pulmonary metastases.

The patient declined treatment with sorafenib due to its high cost and poor condition, opting for symptomatic supportive care instead. He subsequently enrolled in a clinical trial (NCT01972672) and received icaritin (600 mg) twice daily from January 2015 to September 2017 as a first‐line therapy. Additionally, thalidomide was administered orally to provide rapid relief from anorexia, sleep disturbances, pain, and other symptoms of cachexia, starting at 50 mg daily and gradually increasing to 150 mg, with no observed drug intolerance or dose‐limiting toxicity. During the treatment period, the patient's mental state, including anorexia, sleep, and pain, improved. Therefore, thalidomide was continued at 150 mg. Simultaneously, intermittent plasma transfusions, hepatoprotective therapy, antiviral agents, diuretics, antiarrhythmic medications, coronary circulation improvement measures, and other symptomatic and supportive treatments were administered.

**FIGURE 1:**
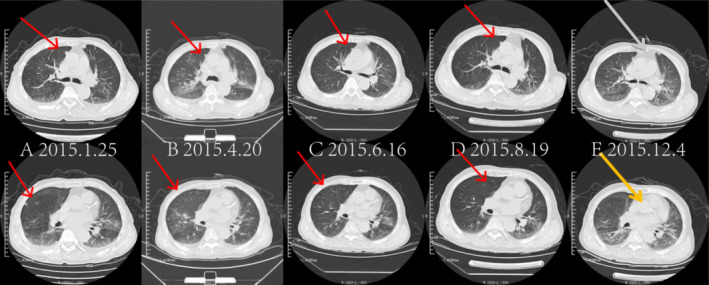
Changes in lung lesions from January 2015 to December 2015: The number of lung metastases significantly decreased, with a substantial reduction in volume and continuous improvement in lesions. Panels A–E show plain chest CT scans of lung tumor metastases, which were nearly in the same plane, gradually decreasing and eventually disappearing between January 25, 2015, and December 4, 2015 (arrows).

On January 25, 2015, the patient's imaging findings showed significant improvement compared to December 2014, with the disappearance of multiple nodular lesions in both lungs and a notable reduction in their size (Figure 1A). Abdominal CT revealed an irregular, hypodense nodular shadow in the liver, along with multiple liver lesions, cirrhosis, splenomegaly, cholecystitis, pelvic and abdominal fluid, and pancreatic atrophy. Notably, AFP levels decreased rapidly to 173 ng/mL after 2 months, and the patient's quality of life improved significantly (Table 1).

**TABLE 1:** Assessment of pulmonary and hepatic efficacy and changes in tumor marker indices during treatment.

Timeline	AFP	CEA	HCV RNA	Lung lesions	Liver lesions
2014.8	466	Normal	17 400	None	Postoperative examination
2014.12	> 2000	Normal	17 600	Double lung metastasis	Intrahepatic metastases
2015.1.25	173	Normal	27 400	PR	SD
2015.3.17	23.9	Normal	23 400	PR	SD
2015.6	Normal	Normal	21 400	PR	PR
2015.8	13.19	Normal	25 400	PR	PR
2015.12	Normal	Normal	27 400	PR	CR
2016.3	Normal	Normal	17 600	PR	CR
2016.7	Normal	Normal	23 400	PR	CR—subtle signs of anomalous lesions in the caudal lobe of the liver
2016.12	Normal	Normal	23 400	PR	Anomalous signs in the right and Caudal lobe
2017.3.14	Normal	Normal	25 400	PR	PD of the right lobe and caudal lobe tumors
2017.7.25	0.88	3.23	26 400	PR	PD
2017.9.6	0.7	3.11	31 340	PR	PD

**FIGURE 2:**
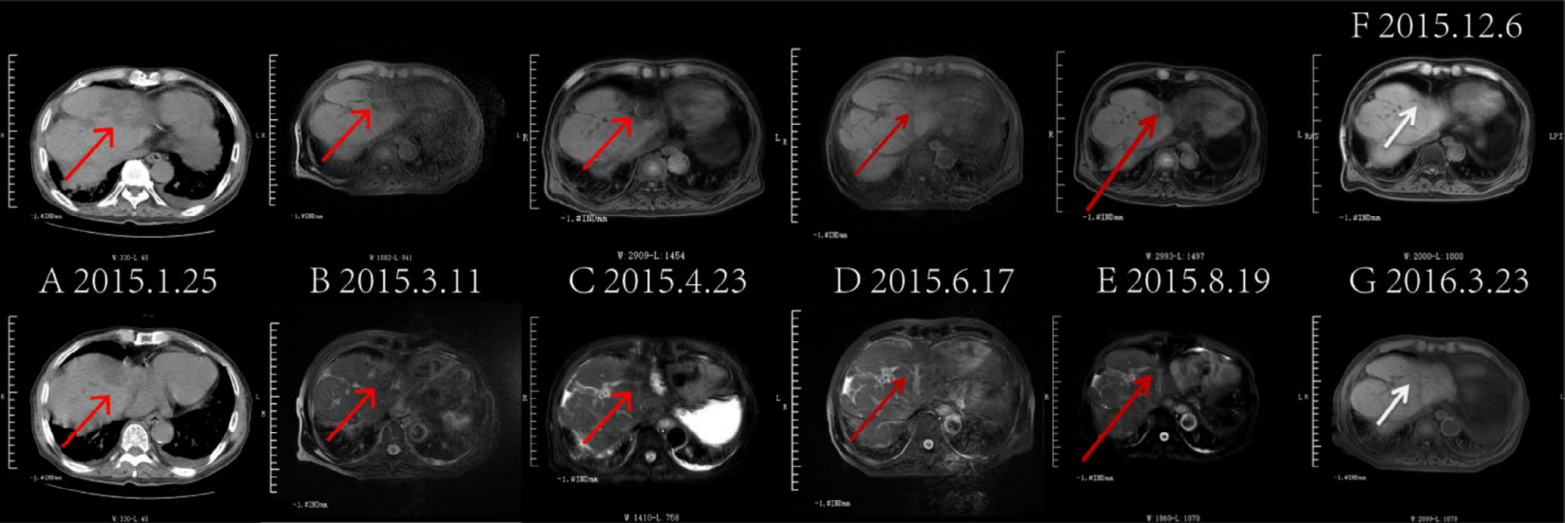
Changes in liver lesions from January 2015 to March 2016: There was a gradual reduction and eventual disappearance of abnormal lesions in the left liver lobe, with continuous improvement observed. Panel A shows an abdominal CT scan with a high‐density occupying lesion in segment 8 of the right lobe of the liver. Panels B–G depict abdominal MRI results, illustrating the tumor's gradual decrease over the treatment period from March 2015 to March 2016. The imaging after 1 year of treatment shows a significant reduction compared to pre‐treatment (arrows).

Follow‐up examinations conducted every 2–3 months showed a significant reduction in lung metastases, with continuous improvement in lesions and sustained PR based on efficacy assessments from January 25 to December 4, 2015 (Figure 1, Table 1). Additionally, abdominal CT and MRI indicated gradual shrinkage and eventual disappearance of abnormal lesions in the left liver lobe (Figure 2). The therapeutic effect of the tumor was evaluated using RECIST 1.1 criteria.

**FIGURE 3:**
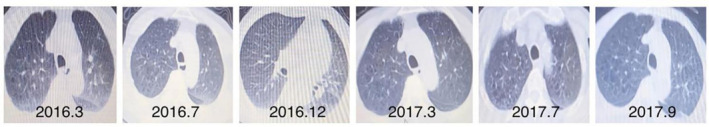
Changes in lung lesions from March 2016 to September 2017: Right lung nodules were significantly smaller than those observed in March 2016; however, the difference was not statistically significant upon follow‐up. A plain chest CT scan displayed multiple metastases in both lungs. Although regular check‐up results showed no significant changes, a comparison of imaging data from March 2016 to September 2017 revealed a notable reduction in the size of tumor metastases following icaritin treatment.

**FIGURE 4:**
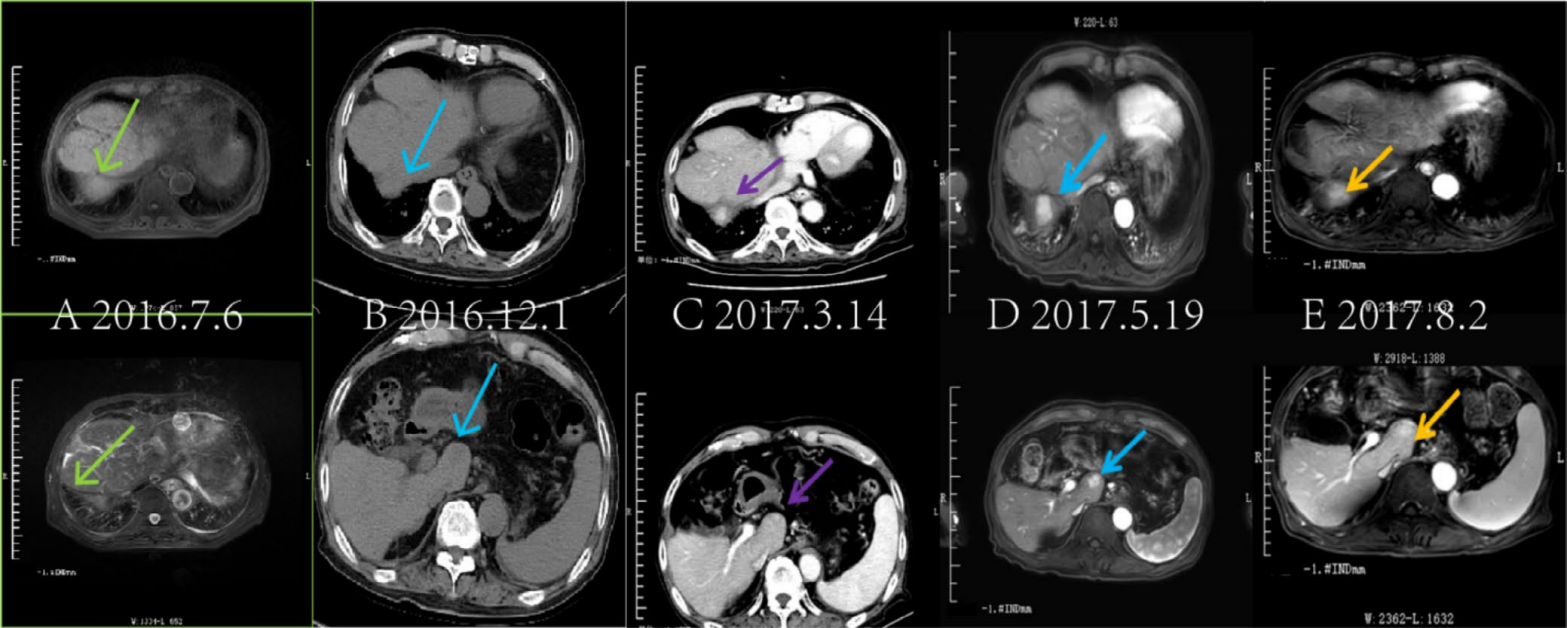
Changes in liver lesions between July 2016 and August 2017 revealed multiple abnormal enhancements in the superior posterior segment of the right and caudal lobes of the liver, which were greater than before. (A) The abdominal MRI shows the tumor‐occupying lesion in the right lobe of the liver. (B) The abdominal CT scan displays the tumor‐occupying lesion in the caudal lobes of the liver. (C) The abdominal enhanced CT scan demonstrates the liver lesions more clearly. (D, E) The abdominal MRI reveals an increase in liver mass compared to before (arrows).

**FIGURE 5:**
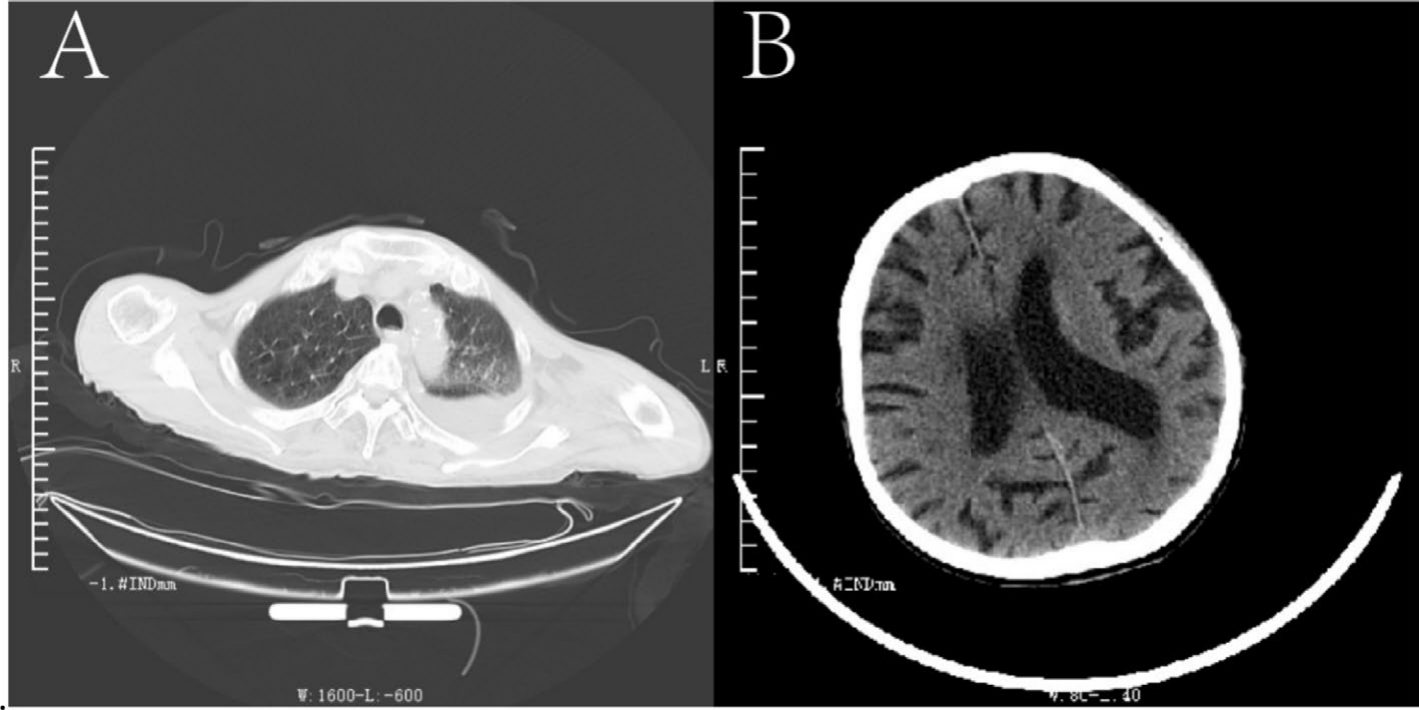
Pneumonia and cerebral infarction on September 19, 2017: (A) The chest CT plain scan shows aspiration pneumonia in both lungs, with a small to moderate amount of pleural effusion in the left lung. (B) The head CT plain scan indicated cerebral infarction in the right temporal lobe in September 2017.

Subsequent CT scans in July 2016 revealed a significant reduction in right pulmonary nodules similar to that observed in March 2016. A comparison of CT results from December 2016 with those from July 2016 showed no significant changes. Additionally, CT identified bilateral lung inflammation, central lobar emphysema, and bilateral pleural effusions, with no substantial changes in nodules noted between March and September 2017. The lung lesions maintained a persistent PR during the efficacy assessments (Figure 3). Between July 2016 and March 2017, possible abnormal lesions were observed in the right and caudal lobes of the liver. On March 14, 2017, plain and enhanced CT scans revealed multiple abnormal enhancements in the superior posterior right and caudal lobes, consistent with imaging manifestations of HCC, despite a normal AFP level. The evaluation of solid tumors in the liver showed progressive disease (PD), with an ECOG‐PS of 2, liver pain rated at 3 on the numerical rating scale (NRS), and ongoing use of oral oxycodone hydrochloride extended‐release tablets for pain relief while continuing treatment with icaritin. CT and MRI findings between March and July 2017 indicated multiple abnormal intensifications in the superior posterior segment of the right and caudal lobes, which were larger than those previously noted. The patient exhibited partial remission of liver lesions, along with cirrhosis, splenomegaly, and ascites (Figure 4). Specific efficacy assessments for pulmonary and hepatic lesions, along with changes in AFP levels, are detailed in Table 1. The patient passed away from aspiration pneumonia and a cerebral infarction in the right temporal lobe in September 2017 (Figure 5).

## 3 Case Summary

**FIGURE 6:**
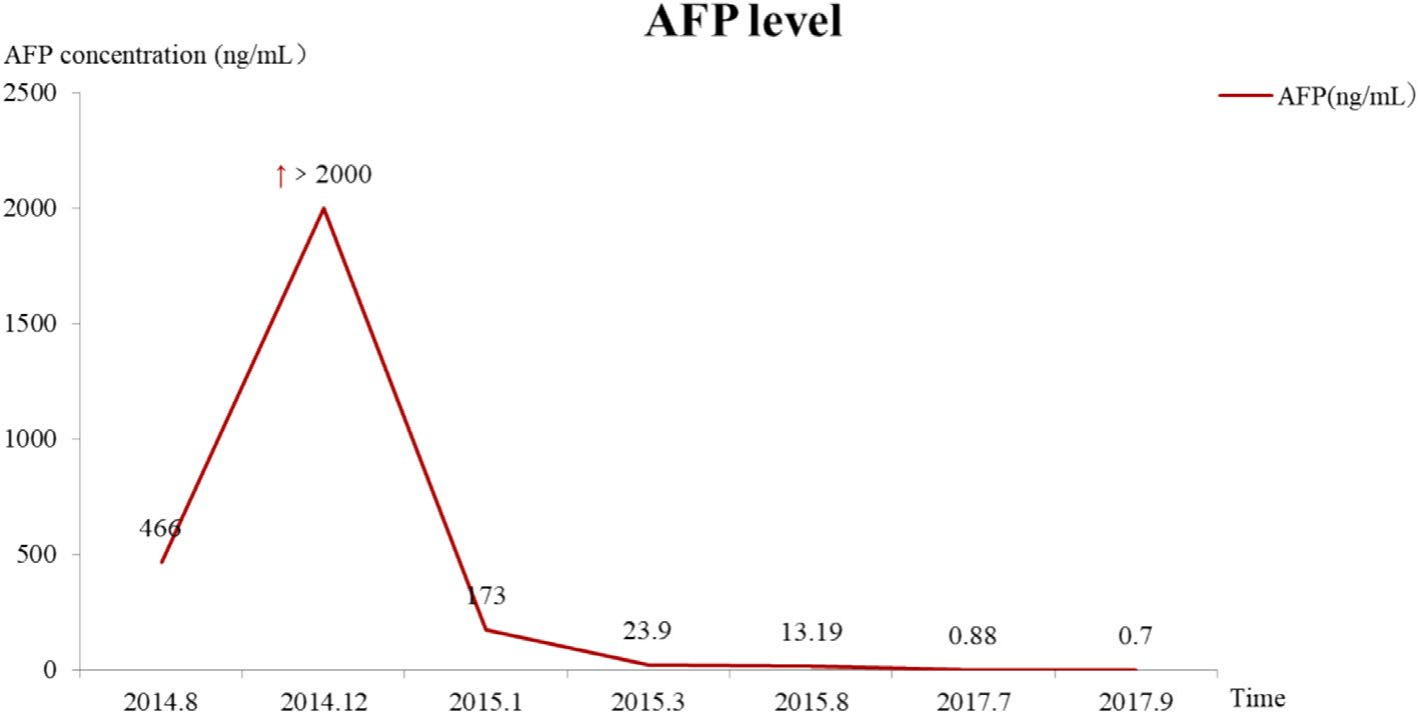
Changes in AFP values during treatment.

The patient was diagnosed with postoperative HCC with bilateral lung metastases, and owing to the lack of suitable treatment, he received icaritin irregularly as postoperative therapy. After recurrence in December 2014, he began treatment with icaritin and thalidomide. As an elderly patient with a suboptimal condition and a very high AFP level at the time of consultation, the patient demonstrated a rapid decrease in AFP levels, a significant reduction in liver and lung lesions, and a considerable, sustained improvement in his condition. After approximately 2 months of treatment, the patient's progression‐free survival (PFS) and overall survival (OS) were 24 and 33 months, respectively, indicating a significant therapeutic effect (Figure 6).

**FIGURE 7:**
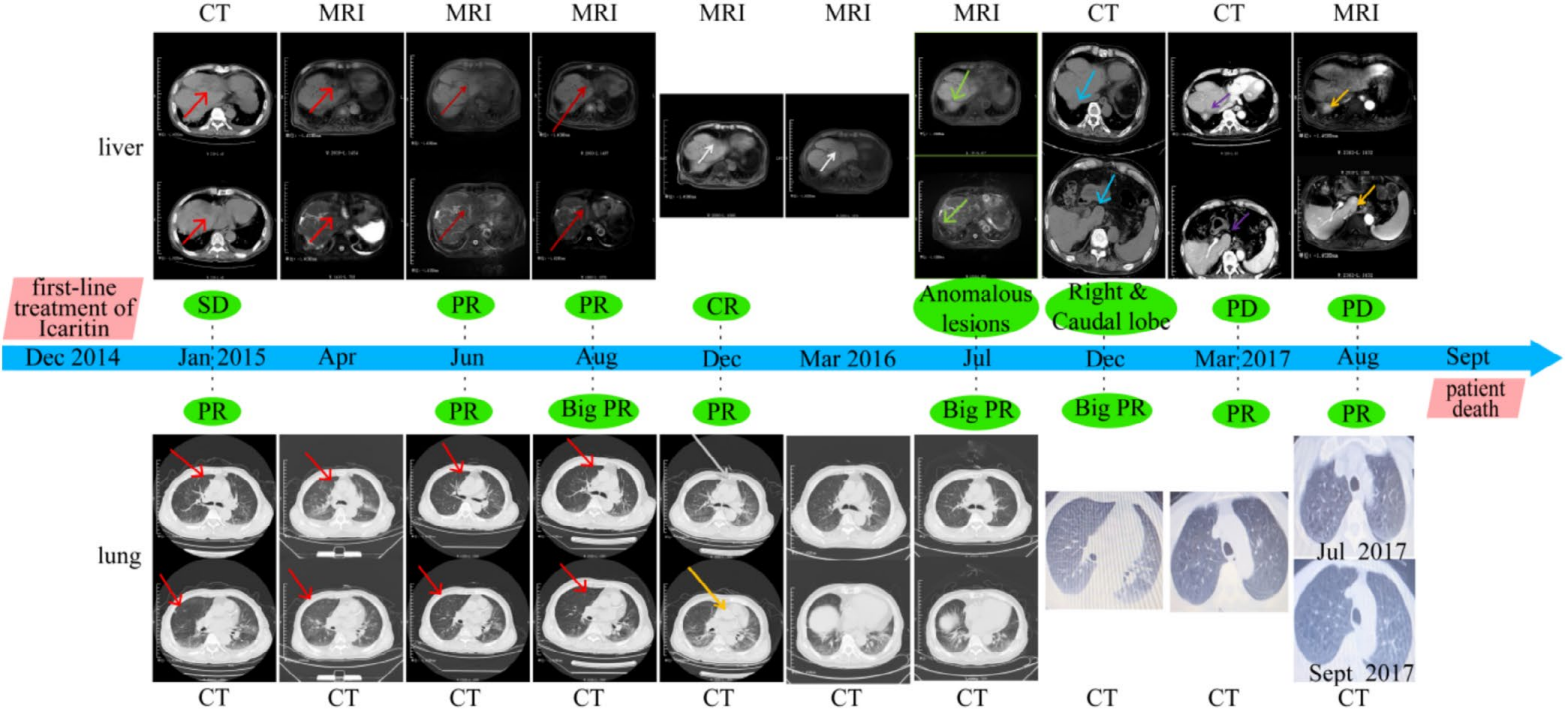
Summary of the treatment process. The imaging at different stages showed changes in the primary liver lesions and bilateral lung metastases after treatment with icaritin.

Additionally, the patient maintained good physical condition during the treatment process, without severe adverse events, and with normal AFP levels. Finally, on September 19, 2017, the patient died of cerebral infarction with aspiration pneumonia instead of tumor progression. This case indicates that the dosage should be sufficient and consistent to obtain the best outcomes with icaritin, and the whole treatment course is summarized in Figure 7.

## 4 Discussion

Currently, icaritin has made significant progress in the systemic treatment of HCC, with various therapeutic options demonstrating exciting potential for HCC treatment. Combination therapies, including VEGF inhibitors, transarterial chemoembolization (TACE), and immune checkpoint inhibitors (ICIs), have demonstrated superior efficacy in the treatment of tumors [14, 15]. The success of studies such as IMBrave150 and ORIENT‐32 has opened a new chapter for applying targeted immune combination therapy in HCC. However, combination therapy with targeted immune agents inevitably exacerbates the adverse effects of drugs, which can lead to grade 3 or higher immune‐related liver damage or hepatitis [16]. In addition, a large number of HCC patients have poor underlying disease statuses, such as advanced age (> 75 years), a lower white blood cell count of 2500–3000, a platelet count > 50 000, mild ascites, an HBV‐DNA level of 500–2000, a liver function score of B or C, a large liver tumor burden, and a cancer thrombus invading the portal vein. As a consequence, sorafenib, lenvatinib, or first‐line combination regimens are inappropriate for these patients based on the Guideline, Diagnosis, Management, and Treatment of HCC [17, 18, 19]. Approximately 70% of patients who met the above criteria were enrolled in the phase III clinical study of icaritin. There is an unmet clinical need for safer and more effective therapeutic options for individuals with advanced HCC. Icaritin may be a more suitable and feasible treatment for these patients as a first‐line alternative to immune combination TKIs or for direct combination with targeted immune, second‐line, or combination therapies.

This patient presented with postoperative HCC and bilateral lung metastases, advanced age, suboptimal health conditions, and high AFP levels. Additionally, the patient had a Child‐Pugh score of B, 8, which, combined with the elevated AFP levels, rendered them unsuitable for sorafenib treatment, as sorafenib is only indicated for Child‐Pugh A or well‐compensated B patients. High AFP levels are also associated with a poor prognosis in patients receiving sorafenib therapy [20, 21]. Thus, a safer and more effective treatment regimen was necessary. After 2 months of combination therapy, the AFP concentration decreased rapidly, and there was a significant reduction in liver and lung lesions. PFS and OS were 24 and 33 months, respectively. Consequently, the patient's overall condition continued to improve rapidly.

Angiogenesis and immunological modulation in the tumor microenvironment may drive these changes. This combination may synergistically normalize tumor blood vessels and stimulate immune activation, leading to positive outcomes. Additionally, patients maintained good mental and physical status, with no significant adverse reactions, highlighting safety benefits and an improved quality of life.

The liver function score is also a key factor in selecting treatment methods for liver cancer. The preservation of hepatic functional reserve is required for postoperative therapy. Previous studies have shown that the worsening of the Child‐Pugh score is an independent predictive factor for poor OS and PFS after treatment with lenvatinib in patients with advanced HCC. Therefore, predicting the hepatic reserve response to TKI therapy is clinically significant because there is a clear correlation between high ANG2 and low VEGF serum levels at baseline and the deterioration of hepatic reserve. In contrast, a phase III clinical study of icaritin revealed that the median deterioration time was 7.3 months, and symptom relief could exceed 6 months for advanced HCC patients, significantly delaying the deterioration of liver function and compensating for the shortcomings of targeted therapy [22]. Therefore, combination therapy with TKIs may further prolong patient survival, improve quality of life, and protect liver function. Cases demonstrating the beneficial effects of icaritin in conjunction with thalidomide are promising for the future treatment of late‐stage HCC with generally poorer conditions. Furthermore, newer methods, such as evidence from the Royal Marsden Hospital (RMH) score and the albumin levels test, may be used to assess patients' prognosis in future cases [23, 24]. Meanwhile, this study has several limitations: (1) It is based on a single case report, limiting generalizability; (2) Additional data are needed to support the combination therapy with icaritin; (3) There is a lack of molecular pathology data, which hinders our understanding of icaritin's mechanism of action in relation to targeted or immunotherapy.

Moreover, we identified that there were certain deficiencies in the initial diagnosis and treatment of this patient. Upon identifying a mass in the right lobe of the liver, a more comprehensive evaluation, including the combination of tumor markers, hepatitis history, and imaging results, should have been conducted to determine the type and stage of the disease, followed by the implementation of an appropriate treatment plan. The detailed rationale for the decisions made at that time is unclear, including why the patient did not undergo surgery earlier, as the patient first presented to our hospital in 2014 following liver resection performed at another institution. However, it is evident that more comprehensive diagnostic evaluations and a more careful decision‐making process would have been necessary. Furthermore, earlier surgical intervention would likely have been more beneficial in controlling the progression of the patient's disease.

Based on the current study, the combination of icaritin, a small‐molecule immunomodulator, with antiangiogenic agents has shown promising results. It can be used in combination with various approaches based on its mechanism of action. For example, systemic antitumor drugs, such as targeted and immune therapies, remain theoretically viable, as well as combined interventional and surgical applications. For instance, combining icaritin with targeted therapies, immunotherapies, other systemic antitumor agents, interventional approaches, or adjuvant therapy after surgery may be feasible [15]. When combined with TACE drugs, icaritin can synergistically induce immunogenic cell death (ICD) by inhibiting the production of HIF‐1α and VEGF. Prior clinical studies have revealed that elevated levels of IL‐6/TNF‐α and AFP are strongly associated with postoperative recurrence [25, 26, 27, 28, 29].

In addition, icaritin has been recommended as the preferred drug for patients with advanced liver cancer who are not suitable for standard treatment in stage IV, according to the China Guidelines for the Diagnosis and Treatment of Primary Liver Cancer (Version 2024) [30]. Our case highlights the efficacy and safety of icaritin in treating advanced HCC with pulmonary metastasis, characterized by reduced AFP levels, diminished tumor size, and minimal side effects. The combination with thalidomide may enhance overall treatment outcomes. This evidence supports icaritin as a viable treatment option, warranting further research into its use in combination with other drugs.

## 5 Conclusion

Icaritin combined with thalidomide as a first‐line treatment achieved PR efficacy and survival benefits in patients with advanced HCC. This therapeutic strategy offers clinicians a potential option to address the treatment gap for patients who cannot tolerate standard therapies due to poor physical conditions. The findings suggest that icaritin's unique immunomodulatory properties and safety profile merit further investigation and consideration in developing treatment protocols for HCC, particularly for patients who are not candidates for more aggressive therapies.

## Author Contributions

H.L.: conceptualization and methodology. Z.J., F.Z., and Z.W.: data analysis, validation, and original draft preparation. Z.J., and H.L.: review and editing, and supervision. All the authors have read and agreed to the published version of the manuscript.

## Consent

Written informed consent was provided by the patient for permission to receive therapy and to publish this case report.

## Conflicts of Interest

The authors declare no conflicts of interest.

## Data Availability

The dataset supporting the conclusions of this article is available upon request. Please contact the corresponding author.
